# Supplementary material for: Investigating the replicability of preclinical cancer biology
Source: eLife. 2021 Dec 10;10:e71601. doi: 10.7554/eLife.71601 (PMC8651293; doi:10.7554/eLife.71601)
Supplement: Supplementary file 1. [file elife-71601-supp1.docx]

**Reproducibility in Cancer Biology: Investigating the replicability of preclinical cancer biology (Errington *et al*. 2021 *eLife* 10:e71601)**

**Supplementary file 1**

*Tables S1–S11*

**Table S1. Replication success rates for each criterion using native effect sizes.**

|  | Papers | Experiments | Effects | All outcomes |
| --- | --- | --- | --- | --- |
| Total number | 23 | 50 | 158 | 188 |
|  |  |  |  |  |
| **ORIGINAL POSITIVE RESULTS** |  |  |  |  |
| ***Numerical results*** |  |  |  |  |
| Same direction | 17 of 19 (89%) | 26 of 35 (74%) | 80 of 101 (79%) | 95 of 116 (82%) |
| Direction and statistical significance | 8 of 19 (42%) | 16 of 33 (48%) | 41 of 97 (42%) | 51 of 112 (46%) |
| Original ES in replication CI | 4 of 19 (21%) | 2 of 33 (6%) | 14 of 97 (14%) | 15 of 112 (13%) |
| Replication ES in original CI | 5 of 19 (26%) | 10 of 33 (30%) | 33 of 97 (34%) | 33 of 112 (29%) |
| Replication ES in PI (*P_orig_*) | 8 of 19 (42%) | 14 of 33 (42%) | 50 of 97 (52%) | 52 of 112 (46%) |
| Replication ES ≥ original ES | 1 of 19 (5%) | 1 of 33 (3%) | 3 of 97 (3%) | 3 of 112 (3%) |
| Meta-analysis (*p* < 0.05) | 16 of 19 (84%) | 26 of 33 (79%) | 62 of 97 (64%) | 77 of 112 (69%) |
| ***Representative images*** |  |  |  |  |
| Same direction | 9 of 10 (90%) | 13 of 16 (81%) | 30 of 35 (86%) | 36 of 45 (80%) |
| Direction and statistical significance | 3 of 8 (38%) | 7 of 12 (58%) | 14 of 22 (64%) | 14 of 22 (64%) |
| Original image in replication CI | 5 of 7 (71%) | 3 of 11 (27%) | 10 of 21 (48%) | 10 of 21 (48%) |
| Replication effect ≥ original image | 3 of 7 (43%) | 5 of 11 (45%) | 8 of 21 (38%) | 8 of 21 (38%) |
| ***Sample sizes*** |  |  |  |  |
| Median [IQR] original sample size | 46.0 [20.0–100] | 20.0 [8.5–48.0] | 8.0 [6.0–13.0] | 8.0 [6.0–18.0] |
| Median [IQR] replication sample size | 50.0 [28.0–128] | 24.0 [11.5–50.0] | 12.0 [8.0–22.2] | 12.0 [8.0–18.0] |
|  |  |  |  |  |
| **ORIGINAL NULL RESULTS** |  |  |  |  |
| ***Numerical results*** |  |  |  |  |
| Same direction | N/A | N/A | N/A | N/A |
| Direction and statistical significance | 8 of 11 (73%) | 9 of 12 (75%) | 10 of 15 (67%) | 11 of 20 (55%) |
| Original ES in replication CI | 6 of 11 (55%) | 7 of 12 (58%) | 9 of 15 (60%) | 8 of 20 (40%) |
| Replication ES in original CI | 9 of 11 (82%) | 10 of 12 (83%) | 12 of 15 (80%) | 11 of 20 (55%) |
| Replication ES in PI (*P_orig_*) | 9 of 11 (82%) | 10 of 12 (83%) | 12 of 15 (80%) | 12 of 20 (60%) |
| Replication ES ≤ original ES | N/A | N/A | N/A | N/A |
| Meta-analysis (*p* > 0.05) | 9 of 11 (82%) | 10 of 12 (83%) | 10 of 15 (67%) | 12 of 20 (60%) |
| ***Representative images*** |  |  |  |  |
| Same direction | N/A | N/A | N/A | N/A |
| Direction and statistical significance | 3 of 3 (100%) | 3 of 3 (100%) | 4 of 5 (80%) | 4 of 5 (80%) |
| Original image in replication CI | 1 of 3 (33%) | 1 of 3 (33%) | 2 of 5 (40%) | 2 of 5 (40%) |
| Replication effect ≤ original image | N/A | N/A | N/A | N/A |
| ***Sample sizes*** |  |  |  |  |
| Median [IQR] original sample size | 16.0 [8.0–25.0] | 12.0 [6.0–20.0] | 15.0 [7.5–31.0] | 18.0 [8.0–514] |
| Median [IQR] replication sample size | 24.0 [16.0–69.0] | 21.0 [8.0–54.0] | 27.0 [8.0–66.8] | 24.0 [16.0–573] |

This table and Tables S2 and S3 are similar to Table 1 in the main article, but use different strategies to aggregate the data. Summary of consistency between original and replication findings separately for original positive results (top) and null results (bottom), and by treating internal replications individually (all outcomes; column 5) and aggregated by effects (column 4), experiments (column 3), and papers (column 2) using native effect sizes (where possible). Effects were meta-analytically combined into experiments with random effect models reflecting the fact that the effects could be heterogeneous within an experiment. And experiments were meta-analytically combined into papers with random effect models for the same reason. All findings coded in terms of consistency with original findings. If original results were null, then a positive result is counted as inconsistent with the original finding. For statistical significance, if original results were interpreted as a positive result but were not statistically significant at *p* < 0.05, then they were treated as a positive result (5 effects), and likewise if they were interpreted as a null result but were statistically significant at *p* < 0.05 they were treated as a null result (3 effects). Also, for statistical significance, for original positive results, replications were successful if they were statistically significant and in the same direction as the original finding; for original null results, replications were successful if they were not statistically significant, regardless of direction. As explained in Table 1, two of the criteria ("same direction" and "Replication ES ≤ original ES") cannot be applied to null results. Mean differences were estimated from the image for original effects based on representative images. Original positive and null effects were kept separate in aggregating into experiments and papers. That is, if a single experiment had both positive and null findings, then the positive findings are summarized in "original positive results" and the null findings are summarized in "original null results". CI = 95% confidence interval; PI = 95% prediction interval; ES = effect size; IQR = interquartile range.

**Table S2. Replication success rates for each criterion summarizing at the effect-level with native effect sizes.**

|  | Papers | Experiments | Effects | All outcomes |
| --- | --- | --- | --- | --- |
| Total number | 23 | 50 | 158 | 188 |
|  |  |  |  |  |
| **ORIGINAL POSITIVE RESULTS** |  |  |  |  |
| ***Numerical results*** |  |  |  |  |
| Same direction | 15 of 19 (79%) | 25 of 35 (71%) | 80 of 101 (79%) | 95 of 116 (82%) |
| Direction and statistical significance | 11 of 19 (58%) | 15 of 33 (45%) | 41 of 97 (42%) | 51 of 112 (46%) |
| Original ES in replication CI | 3 of 19 (16%) | 3 of 33 (9%) | 14 of 97 (14%) | 15 of 112 (13%) |
| Replication ES in original CI | 5 of 19 (26%) | 11 of 33 (33%) | 33 of 97 (34%) | 33 of 112 (29%) |
| Replication ES in PI (P_orig_) | 11 of 19 (58%) | 16 of 33 (48%) | 50 of 97 (52%) | 52 of 112 (46%) |
| Replication ES ≥ original ES | 0 of 19 (0%) | 1 of 33 (3%) | 3 of 97 (3%) | 3 of 112 (3%) |
| Meta-analysis (*p* < 0.05) | 15 of 19 (79%) | 24 of 33 (73%) | 62 of 97 (64%) | 77 of 112 (69%) |
| ***Representative images*** |  |  |  |  |
| Same direction | 8 of 10 (80%) | 13 of 16 (81%) | 30 of 35 (86%) | 36 of 45 (80%) |
| Direction and statistical significance | 4 of 8 (50%) | 8 of 12 (67%) | 14 of 22 (64%) | 14 of 22 (64%) |
| Original image in replication CI | 3 of 7 (43%) | 4 of 11 (36%) | 10 of 21 (48%) | 10 of 21 (48%) |
| Replication effect ≥ original image | 1 of 7 (14%) | 5 of 11 (45%) | 8 of 21 (38%) | 8 of 21 (38%) |
| ***Sample sizes*** |  |  |  |  |
| Median [IQR] original sample size | 46.0 [20.0–100] | 20.0 [8.5–48.0] | 8.0 [6.0–13.0] | 8.0 [6.0–18.0] |
| Median [IQR] replication sample size | 50.0 [28.0–128] | 24.0 [11.5–50.0] | 12.0 [8.0–22.2] | 12.0 [8.0–18.0] |
|  |  |  |  |  |
| **ORIGINAL NULL RESULTS** |  |  |  |  |
| ***Numerical results*** |  |  |  |  |
| Same direction | N/A | N/A | N/A | N/A |
| Direction and statistical significance | 8 of 11 (73%) | 9 of 12 (75%) | 10 of 15 (67%) | 11 of 20 (55%) |
| Original ES in replication CI | 6 of 11 (55%) | 7 of 12 (58%) | 9 of 15 (60%) | 8 of 20 (40%) |
| Replication ES in original CI | 9 of 11 (82%) | 10 of 12 (83%) | 12 of 15 (80%) | 11 of 20 (55%) |
| Replication ES in PI (P_orig_) | 9 of 11 (82%) | 10 of 12 (83%) | 12 of 15 (80%) | 12 of 20 (60%) |
| Replication ES ≤ original ES | N/A | N/A | N/A | N/A |
| Meta-analysis (*p* > 0.05) | 8 of 11 (73%) | 9 of 12 (75%) | 10 of 15 (67%) | 12 of 20 (60%) |
| ***Representative images*** |  |  |  |  |
| Same direction | N/A | N/A | N/A | N/A |
| Direction and statistical significance | 2 of 3 (67%) | 2 of 3 (67%) | 4 of 5 (80%) | 4 of 5 (80%) |
| Original image in replication CI | 1 of 3 (33%) | 1 of 3 (33%) | 2 of 5 (40%) | 2 of 5 (40%) |
| Replication effect ≤ original image | N/A | N/A | N/A | N/A |
| ***Sample sizes*** |  |  |  |  |
| Median [IQR] original sample size | 16.0 [8.0–25.0] | 12.0 [6.0–20.0] | 15.0 [7.5–31.0] | 18.0 [8.0–514] |
| Median [IQR] replication sample size | 24.0 [16.0–69.0] | 21.0 [8.0–54.0] | 27.0 [8.0–66.8] | 24.0 [16.0–573] |

Summary of consistency between original and replication findings separately for original positive results (top) and null results (bottom), and by treating internal replications individually (all outcomes; column 5) and summarized by effects (column 4), experiments (column 3), and papers (column 2) using native effect sizes (where possible). Summarized effect-level metrics at the experiment and paper level were obtained by calculating percentages to summarize binary metrics of replication success, and by using harmonic mean *p*-values to summarize the continuous metric. All findings coded in terms of consistency with original findings: see the caption of Table S1 for details.

**Table S3. Replication success rates for each criterion summarizing at the effect-level with SMD effect sizes.**

|  | Papers | Experiments | Effects | All outcomes |
| --- | --- | --- | --- | --- |
| Total number | 23 | 50 | 158 | 188 |
|  |  |  |  |  |
| **ORIGINAL POSITIVE RESULTS** |  |  |  |  |
| ***Numerical results*** |  |  |  |  |
| Same direction | 15 of 19 (79%) | 25 of 35 (71%) | 80 of 101 (79%) | 95 of 116 (82%) |
| Direction and statistical significance | 11 of 19 (58%) | 16 of 33 (48%) | 42 of 97 (43%) | 44 of 112 (39%) |
| Original ES in replication CI | 3 of 19 (16%) | 4 of 33 (12%) | 17 of 97 (18%) | 26 of 112 (23%) |
| Replication ES in original CI | 7 of 19 (37%) | 14 of 33 (42%) | 42 of 97 (43%) | 50 of 112 (45%) |
| Replication ES in PI (P_orig_) | 10 of 19 (53%) | 17 of 33 (52%) | 56 of 97 (58%) | 67 of 112 (60%) |
| Replication ES ≥ original ES | 0 of 19 (0%) | 1 of 33 (3%) | 3 of 97 (3%) | 3 of 112 (3%) |
| Meta-analysis (*p* < 0.05) | 15 of 19 (79%) | 24 of 33 (73%) | 60 of 97 (62%) | 75 of 112 (67%) |
| ***Representative images*** |  |  |  |  |
| Same direction | 7 of 10 (70%) | 12 of 16 (75%) | 28 of 35 (80%) | 34 of 45 (76%) |
| Direction and statistical significance | 4 of 8 (50%) | 8 of 12 (67%) | 14 of 22 (64%) | 14 of 22 (64%) |
| Original image in replication CI | 3 of 7 (43%) | 4 of 11 (36%) | 10 of 21 (48%) | 10 of 21 (48%) |
| Replication effect ≥ original image | 1 of 7 (14%) | 5 of 11 (45%) | 7 of 21 (33%) | 7 of 21 (33%) |
| ***Sample sizes*** |  |  |  |  |
| Median [IQR] original sample size | 46.0 [20.0–100] | 20.0 [8.5–48.0] | 8.0 [6.0–13.0] | 8.0 [6.0–18.0] |
| Median [IQR] replication sample size | 50.0 [28.0–128] | 24.0 [11.5–50.0] | 12.0 [8.0–22.2] | 12.0 [8.0–18.0] |
|  |  |  |  |  |
| **ORIGINAL NULL RESULTS** |  |  |  |  |
| ***Numerical results*** |  |  |  |  |
| Same direction | N/A | N/A | N/A | N/A |
| Direction and statistical significance | 9 of 11 (82%) | 10 of 12 (83%) | 11 of 15 (73%) | 10 of 20 (50%) |
| Original ES in replication CI | 8 of 11 (73%) | 9 of 12 (75%) | 11 of 15 (73%) | 12 of 20 (60%) |
| Replication ES in original CI | 9 of 11 (82%) | 10 of 12 (83%) | 12 of 15 (80%) | 13 of 20 (65%) |
| Replication ES in PI (P_orig_) | 9 of 11 (82%) | 10 of 12 (83%) | 12 of 15 (80%) | 14 of 20 (70%) |
| Replication ES ≤ original ES | N/A | N/A | N/A | N/A |
| Meta-analysis (*p* > 0.05) | 9 of 11 (82%) | 10 of 12 (83%) | 10 of 15 (67%) | 11 of 20 (55%) |
| ***Representative images*** |  |  |  |  |
| Same direction | N/A | N/A | N/A | N/A |
| Direction and statistical significance | 2 of 3 (67%) | 2 of 3 (67%) | 4 of 5 (80%) | 4 of 5 (80%) |
| Original image in replication CI | 1 of 3 (33%) | 1 of 3 (33%) | 3 of 5 (60%) | 3 of 5 (60%) |
| Replication effect ≤ original image | N/A | N/A | N/A | N/A |
| ***Sample sizes*** |  |  |  |  |
| Median [IQR] original sample size | 16.0 [8.0–25.0] | 12.0 [6.0–20.0] | 15.0 [7.5–31.0] | 18.0 [8.0–514] |
| Median [IQR] replication sample size | 24.0 [16.0–69.0] | 21.0 [8.0–54.0] | 27.0 [8.0–66.8] | 24.0 [16.0–573] |

Summary of consistency between original and replication findings separately for original positive results (top) and null results (bottom), and by treating internal replications individually (all outcomes; column 5) and summarized by effects (column 4), experiments (column 3), and papers (column 2) using standardized mean difference (SMD) scale. Summarized effect-level metrics at the experiment and paper level were obtained by calculating percentages to summarize binary metrics of replication success, and by using harmonic mean *p*-values to summarize the continuous metric. All findings coded in terms of consistency with original findings: see the caption of Table S1 for details.

**Table S4. Pattern of replication success and failure across three significance testing criteria using native effect sizes.**

|  | Papers | | Experiments | | Effects | | All outcomes | |
| --- | --- | --- | --- | --- | --- | --- | --- | --- |
| Total number | 23 | | 50 | | 158 | | 188 | |
|  |  |  |  |  |  |  |  |  |
| **ORIGINAL POSITIVE RESULTS** |  |  |  |  |  |  |  |  |
| Succeeded on all three criteria | 1 | 5% | 1 | 3% | 11 | 11% | 12 | 11% |
| [1] Failed only on significance and direction | 1 | 5% | 0 | 0% | 2 | 2% | 2 | 2% |
| [2] Failed only on original in replication confidence interval | 2 | 11% | 5 | 15% | 10 | 10% | 10 | 9% |
| [3] Failed only on replication in original confidence interval | 1 | 5% | 1 | 3% | 1 | 1% | 1 | 1% |
| Failed only on [1] and [2] | 1 | 5% | 4 | 12% | 10 | 10% | 9 | 8% |
| Failed only on [2] and [3] | 4 | 21% | 9 | 27% | 19 | 20% | 28 | 25% |
| Failed only on [1] and [3] | 1 | 5% | 0 | 0% | 0 | 0% | 0 | 0% |
| Failed on all three criteria [1], [2], and [3] | 8 | 42% | 13 | 39% | 44 | 45% | 50 | 45% |
| Total | 19 |  | 33 |  | 97 |  | 112 |  |
|  |  |  |  |  |  |  |  |  |
| **ORIGINAL NULL RESULTS** |  |  |  |  |  |  |  |  |
| Succeeded on all three criteria | 5 | 45% | 6 | 50% | 7 | 47% | 7 | 35% |
| [1] Failed only on significance and direction | 1 | 9% | 1 | 8% | 2 | 13% | 1 | 5% |
| [2] Failed only on original in replication confidence interval | 1 | 9% | 1 | 8% | 1 | 7% | 1 | 5% |
| [3] Failed only on replication in original confidence interval | 0 | 0% | 0 | 0% | 0 | 0% | 0 | 0% |
| Failed only on [1] and [2] | 2 | 18% | 2 | 17% | 2 | 13% | 2 | 10% |
| Failed only on [2] and [3] | 2 | 18% | 2 | 17% | 2 | 13% | 3 | 15% |
| Failed only on [1] and [3] | 0 | 0% | 0 | 0% | 0 | 0% | 0 | 0% |
| Failed on all three criteria [1], [2], and [3] | 0 | 0% | 0 | 0% | 1 | 7% | 6 | 30% |
| Total | 11 |  | 12 |  | 15 |  | 20 |  |

This table and Tables S5 and S6 are similar to Table 2 in the main article, but use different strategies to aggregate the data. Number of replications that succeeded or failed to replicate results in original experiments according to three criteria within the null hypothesis significance testing framework, using native effect sizes (where possible): statistical significance (*p* < 0.05) and same direction; original effect size inside 95% confidence interval of replication effect size; replication effect size inside 95% confidence interval of original effect size. Data for original positive results and original null results are shown separately, as are data for all outcomes and aggregated by effect, experiment, and paper. Effects were meta-analytically combined into experiments with random effect models to reflect the fact that the effects could be heterogeneous within an experiment, and experiments were meta-analytically combined into papers with random effect models for the same reason.

**Table S5. Pattern of replication success and failure across three significance testing criteria summarizing at the effect-level with native effect sizes.**

|  | Papers | | Experiments | | Effects | | All outcomes | |
| --- | --- | --- | --- | --- | --- | --- | --- | --- |
| Total number | 23 | | 50 | | 158 | | 188 | |
|  |  |  |  |  |  |  |  |  |
| **ORIGINAL POSITIVE RESULTS** |  |  |  |  |  |  |  |  |
| Succeeded on all three criteria | 2 | 11% | 2 | 6% | 11 | 11% | 12 | 11% |
| [1] Failed only on significance and direction | 0 | 0% | 0 | 0% | 2 | 2% | 2 | 2% |
| [2] Failed only on original in replication confidence interval | 2 | 11% | 6 | 18% | 10 | 10% | 10 | 9% |
| [3] Failed only on replication in original confidence interval | 1 | 5% | 1 | 3% | 1 | 1% | 1 | 1% |
| Failed only on [1] and [2] | 1 | 5% | 3 | 9% | 10 | 10% | 9 | 8% |
| Failed only on [2] and [3] | 6 | 32% | 6 | 18% | 19 | 20% | 28 | 25% |
| Failed only on [1] and [3] | 0 | 0% | 0 | 0% | 0 | 0% | 0 | 0% |
| Failed on all three criteria [1], [2], and [3] | 7 | 37% | 15 | 45% | 44 | 45% | 50 | 45% |
| Total | 19 |  | 33 |  | 97 |  | 112 |  |
|  |  |  |  |  |  |  |  |  |
| **ORIGINAL NULL RESULTS** |  |  |  |  |  |  |  |  |
| Succeeded on all three criteria | 5 | 45% | 6 | 50% | 7 | 47% | 7 | 35% |
| [1] Failed only on significance and direction | 1 | 9% | 1 | 8% | 2 | 13% | 1 | 5% |
| [2] Failed only on original in replication confidence interval | 1 | 9% | 1 | 8% | 1 | 7% | 1 | 5% |
| [3] Failed only on replication in original confidence interval | 0 | 0% | 0 | 0% | 0 | 0% | 0 | 0% |
| Failed only on [1] and [2] | 2 | 18% | 2 | 17% | 2 | 13% | 2 | 10% |
| Failed only on [2] and [3] | 2 | 18% | 2 | 17% | 2 | 13% | 3 | 15% |
| Failed only on [1] and [3] | 0 | 0% | 0 | 0% | 0 | 0% | 0 | 0% |
| Failed on all three criteria [1], [2], and [3] | 0 | 0% | 0 | 0% | 1 | 7% | 6 | 30% |
| Total | 11 |  | 12 |  | 15 |  | 20 |  |

Number of replications that succeeded or failed to replicate results in original experiments according to three criteria within the null hypothesis significance testing framework, using native effect sizes (where possible): statistical significance (*p* < 0.05) and same direction; original effect size inside 95% confidence interval of replication effect size; replication effect size inside 95% confidence interval of original effect size. Data for original positive results and original null results are shown separately, as are data for all outcomes and aggregated by effect, experiment, and paper. Summarized effect-level metrics at the experiment and paper level were obtained by calculating percentages to summarize binary metrics of replication success, and by using harmonic mean *p*-values to summarize the continuous metric.

**Table S6. Pattern of replication success and failure across three significance testing criteria summarizing at the effect-level with SMD effect sizes.**

|  | Papers | | Experiments | | Effects | | All outcomes | |
| --- | --- | --- | --- | --- | --- | --- | --- | --- |
| Total number | 23 | | 50 | | 158 | | 188 | |
|  |  |  |  |  |  |  |  |  |
| **ORIGINAL POSITIVE RESULTS** |  |  |  |  |  |  |  |  |
| Succeeded on all three criteria | 3 | 16% | 3 | 9% | 13 | 13% | 20 | 18% |
| [1] Failed only on significance and direction | 0 | 0% | 1 | 3% | 4 | 4% | 6 | 5% |
| [2] Failed only on original in replication confidence interval | 3 | 16% | 8 | 24% | 14 | 14% | 10 | 9% |
| [3] Failed only on replication in original confidence interval | 0 | 0% | 0 | 0% | 0 | 0% | 0 | 0% |
| Failed only on [1] and [2] | 1 | 5% | 2 | 6% | 11 | 11% | 14 | 13% |
| Failed only on [2] and [3] | 5 | 26% | 5 | 15% | 15 | 15% | 14 | 13% |
| Failed only on [1] and [3] | 0 | 0% | 0 | 0% | 0 | 0% | 0 | 0% |
| Failed on all three criteria [1], [2], and [3] | 7 | 37% | 14 | 42% | 40 | 41% | 48 | 43% |
| Total | 19 |  | 33 |  | 97 |  | 112 |  |
|  |  |  |  |  |  |  |  |  |
| **ORIGINAL NULL RESULTS** |  |  |  |  |  |  |  |  |
| Succeeded on all three criteria | 6 | 55% | 7 | 58% | 8 | 53% | 7 | 35% |
| [1] Failed only on significance and direction | 2 | 18% | 2 | 17% | 3 | 20% | 5 | 25% |
| [2] Failed only on original in replication confidence interval | 1 | 9% | 1 | 8% | 1 | 7% | 1 | 5% |
| [3] Failed only on replication in original confidence interval | 0 | 0% | 0 | 0% | 0 | 0% | 0 | 0% |
| Failed only on [1] and [2] | 0 | 0% | 0 | 0% | 0 | 0% | 0 | 0% |
| Failed only on [2] and [3] | 2 | 18% | 2 | 17% | 2 | 13% | 2 | 10% |
| Failed only on [1] and [3] | 0 | 0% | 0 | 0% | 0 | 0% | 0 | 0% |
| Failed on all three criteria [1], [2], and [3] | 0 | 0% | 0 | 0% | 1 | 7% | 5 | 25% |
| Total | 11 |  | 12 |  | 15 |  | 20 |  |

Number of replications that succeeded or failed to replicate results in original experiments according to three criteria within the null hypothesis significance testing framework, using standardized mean difference (SMD) scale: statistical significance (*p* < 0.05) and same direction; original effect size inside 95% confidence interval of replication effect size; replication effect size inside 95% confidence interval of original effect size. Data for original positive results and original null results are shown separately, as are data for all outcomes and aggregated by effect, experiment, and paper. Summarized effect-level metrics at the experiment and paper level were obtained by calculating percentages to summarize binary metrics of replication success, and by using harmonic mean *p*-values to summarize the continuous metric.

**Table S7. Multi-level models testing the association of five moderators with replicability.**

|  |  |  |  | Linear Mixed Model | | | Linear Mixed Model with Imputed Within-Pair Heterogeneity (Sensitivity Analysis) | | |
| --- | --- | --- | --- | --- | --- | --- | --- | --- | --- |
| Replication metric | Model | Coefficient |  | Estimate [95% CI] | *p*-value | *p*-value (Bonferroni) | Estimate [95% CI] | *p*-value | *p*-value (Bonferroni) |
| Mean difference in ES | RMA | Intercept |  | 0.41 [-1.15, 1.97] | 0.55 | 1.00 |  |  |  |
|  | RMA | Animal expt |  | 0.38 [-0.94, 1.69] | 0.54 | 1.00 |  |  |  |
|  | RMA | CRO lab |  | 0.51 [-0.72, 1.74] | 0.34 | 1.00 |  |  |  |
|  | RMA | Core lab |  | -0.24 [-1.95, 1.47] | 0.74 | 1.00 |  |  |  |
|  | RMA | Materials shared |  | -0.45 [-3.70, 2.79] | 0.74 | 1.00 |  |  |  |
|  | RMA | Materials not requested |  | -0.62 [-2.17, 0.93] | 0.36 | 1.00 |  |  |  |
|  | RMA | Clarification quality |  | 0.19 [-0.30, 0.68] | 0.38 | 1.00 |  |  |  |
| Meta-analysis ES | RMA | Intercept |  | 2.37 [0.17, 4.58] | 4E-02 | 0.19 |  |  |  |
|  | RMA | Animal expt |  | -0.3 [-1.42, 0.83] | 0.57 | 1.00 |  |  |  |
|  | RMA | CRO lab |  | 0.36 [-1.15, 1.87] | 0.57 | 1.00 |  |  |  |
|  | RMA | Core lab |  | -0.51 [-1.78, 0.76] | 0.35 | 1.00 |  |  |  |
|  | RMA | Materials shared |  | -1.33 [-4.09, 1.43] | 0.28 | 1.00 |  |  |  |
|  | RMA | Materials not requested |  | -1.38 [-3.61, 0.85] | 0.18 | 0.91 |  |  |  |
|  | RMA | Clarification quality |  | -0.12 [-0.50, 0.26] | 0.48 | 1.00 |  |  |  |
| Replication ES in PI | LMM | Intercept |  | 0.86 [0.38, 1.34] | 4E-03 | 0.02 | 1.06 [0.56, 1.57] | 2E-03 | 9E-03 |
|  | LMM | Animal expt |  | -0.05 [-0.30, 0.20] | 0.67 | 1.00 | 0.09 [-0.24, 0.43] | 0.54 | 1.00 |
|  | LMM | CRO lab |  | 0.11 [-0.35, 0.57] | 0.57 | 1.00 | -0.14 [-0.64, 0.35] | 0.49 | 1.00 |
|  | LMM | Core lab |  | -0.04 [-0.64, 0.56] | 0.86 | 1.00 | -0.25 [-0.89, 0.39] | 0.36 | 1.00 |
|  | LMM | Materials shared |  | -0.43 [-1.22, 0.37] | 0.24 | 1.00 | -0.33 [-1.07, 0.42] | 0.32 | 1.00 |
|  | LMM | Materials not requested |  | -0.2 [-0.71, 0.32] | 0.40 | 1.00 | -0.22 [-0.64, 0.19] | 0.24 | 1.00 |
|  | LMM | Clarification quality |  | -0.02 [-0.16, 0.13] | 0.81 | 1.00 | 0.01 [-0.13, 0.14] | 0.93 | 1.00 |
| *P*_orig_ | LMM | intercept |  | 0.31 [0.06, 0.56] | 2E-02 | 0.12 | 0.38 [0.12, 0.64] | 1E-02 | 0.05 |
|  | LMM | Animal expt |  | -0.03 [-0.17, 0.11] | 0.64 | 1.00 | -0.02 [-0.18, 0.14] | 0.78 | 1.00 |
|  | LMM | CRO lab |  | -0.01 [-0.17, 0.16] | 0.93 | 1.00 | -0.06 [-0.24, 0.12] | 0.45 | 1.00 |
|  | LMM | Core lab |  | -0.09 [-0.35, 0.16] | 0.39 | 1.00 | -0.12 [-0.39, 0.14] | 0.28 | 1.00 |
|  | LMM | Materials shared |  | -0.08 [-0.37, 0.21] | 0.54 | 1.00 | -0.06 [-0.40, 0.27] | 0.66 | 1.00 |
|  | LMM | Materials not requested |  | 0 [-0.27, 0.26] | 0.98 | 1.00 | -0.02 [-0.32, 0.27] | 0.85 | 1.00 |
|  | LMM | Clarification quality |  | -0.01 [-0.07, 0.04] | 0.59 | 1.00 | -0.01 [-0.07, 0.05] | 0.69 | 1.00 |
| Direction and statistically significant | LMM | intercept |  | 0.87 [0.16, 1.58] | 2E-02 | 0.12 |  |  |  |
|  | LMM | Animal expt |  | -0.52 [-0.86, -0.18] | 7E-03 | 0.03 |  |  |  |
|  | LMM | CRO lab |  | -0.2 [-0.63, 0.24] | 0.30 | 1.00 |  |  |  |
|  | LMM | Core lab |  | -0.22 [-0.57, 0.14] | 0.15 | 0.75 |  |  |  |
|  | LMM | Materials shared |  | 0.32 [-0.46, 1.10] | 0.37 | 1.00 |  |  |  |
|  | LMM | Materials not requested |  | 0.4 [-0.29, 1.10] | 0.22 | 1.00 |  |  |  |
|  | LMM | Clarification quality |  | -0.09 [-0.20, 0.02] | 9E-02 | 0.43 |  |  |  |

We used multi-level models to test if five candidate moderators were associated with the replication rates at the effects level for five outcomes (listed in column 1) for the 97 original positive effects with replication pairs. The five moderators were: i) animal experiments vs. non-animal (i.e., *in vitro*) experiments (Animal expt); ii) the use of contract research organizations to conduct replications (CRO lab); iii) the use of academic research core facilities to conduct replications (Core lab); iv) whether the original authors shared materials with the replicating labs (Materials shared; Materials not requested**)**; v) the quality of methodological clarifications made by the original authors upon request from the replicating labs (Clarifications quality); see Methods for more details. The five moderators were included simultaneously in a model predicting replication success, but none of the five showed a consistent, significant association with replication rate, though they were correlated to different extents (see Figure 4). For outcomes that are continuous (e.g., Mean difference in ES), the estimate represents the mean difference between pairs that had a given moderator and pairs that did not have that moderator, holding constant the other moderators. For binary outcomes (e.g., Replication ES in PI), the estimate is the difference in the probability of the outcome for pairs that had the moderator and pairs that did not have the moderator. The models were a linear mixed model for metrics without variances (LMM) and a random-effects meta-analysis for metrics with variances (RMA). We also repeated the analysis with imputed heterogeneity estimates for two outcomes (Replication ES in PI, and P_orig_). Estimate [95% CI] = point estimate for moderation with 95% CI. *p*-value = uncorrected *p*-value; *p*-value (Bonferroni) = *p*-value corrected for multiple testing within, but not across, outcome via Bonferroni (i.e., counting the number of moderators); ES = effect size; PI = prediction interval.

**Table S8. Number of replications inconsistent with original experiments across five replication criteria that render dichotomous outcome assessments using native effect sizes.**

|  | Papers | | Experiments | | Effects | | All outcomes | |
| --- | --- | --- | --- | --- | --- | --- | --- | --- |
|  |  |  |  |  |  |  |  |  |
| **ORIGINAL POSITIVE RESULTS** |  |  |  |  |  |  |  |  |
| Successful replication on all 5 criteria | 1 | 5% | 1 | 3% | 11 | 11% | 12 | 11% |
| Success on 4, Failure on 1 | 4 | 21% | 6 | 18% | 12 | 12% | 12 | 11% |
| Success on 3, Failure on 2 | 1 | 5% | 1 | 3% | 8 | 8% | 7 | 6% |
| Success on 2, Failure on 3 | 6 | 32% | 14 | 42% | 28 | 29% | 39 | 35% |
| Success on 1, Failure on 4 | 5 | 26% | 7 | 21% | 17 | 18% | 21 | 19% |
| Failure to replicate on all 5 criteria | 2 | 11% | 4 | 12% | 21 | 22% | 21 | 19% |
| Total | 19 |  | 33 |  | 97 |  | 112 |  |
|  |  |  |  |  |  |  |  |  |
| **ORIGINAL NULL RESULTS** |  |  |  |  |  |  |  |  |
| Successful replication on all 5 criteria | 5 | 45% | 6 | 50% | 6 | 40% | 6 | 30% |
| Success on 4, Failure on 1 | 1 | 9% | 1 | 8% | 2 | 13% | 2 | 10% |
| Success on 3, Failure on 2 | 2 | 18% | 2 | 17% | 3 | 20% | 1 | 5% |
| Success on 2, Failure on 3 | 3 | 27% | 3 | 25% | 3 | 20% | 4 | 20% |
| Success on 1, Failure on 4 | 0 | 0% | 0 | 0% | 0 | 0% | 5 | 25% |
| Failure to replicate on all 5 criteria | 0 | 0% | 0 | 0% | 1 | 7% | 2 | 10% |
| Total | 11 |  | 12 |  | 15 |  | 20 |  |

This table and Tables S9 and S10 are similar to Table 6 in the main article, but use different strategies to aggregate the data. Five of the criteria we used to assess replications could be used for both positive results and null results. The number of papers, experiments, effects, and outcomes where replications were successful on various numbers of these criteria are shown for positive results (top) and null results (bottom). The five criteria were: i) direction and statistical significance (*p* < 0.05); ii) original effect size in replication 95% confidence interval; iii) replication effect size in original 95% confidence interval; iv) replication effect size in original 95% prediction interval; v) meta-analysis combining original and replication effect sizes is statistically significant (*p* < 0.05). The data in this table are based on native effect sizes (where possible). Effects were meta-analytically combined into experiments with random effect models to reflect the fact that the effects could be heterogeneous within an experiment, and experiments were meta-analytically combined into papers with random effect models for the same reason.

**Table S9. Number of replications inconsistent with original experiments across five replication criteria that render dichotomous outcome assessments summarizing at the effect-level with native effect sizes.**

|  | Papers | | Experiments | | Effects | | All outcomes | |
| --- | --- | --- | --- | --- | --- | --- | --- | --- |
|  |  |  |  |  |  |  |  |  |
| **ORIGINAL POSITIVE RESULTS** |  |  |  |  |  |  |  |  |
| Successful replication on all five criteria | 2 | 11% | 2 | 6% | 11 | 11% | 12 | 11% |
| Success on 4, Failure on 1 | 3 | 16% | 7 | 21% | 12 | 12% | 12 | 11% |
| Success on 3, Failure on 2 | 2 | 11% | 1 | 3% | 8 | 8% | 7 | 6% |
| Success on 2, Failure on 3 | 7 | 37% | 10 | 30% | 28 | 29% | 39 | 35% |
| Success on 1, Failure on 4 | 3 | 16% | 8 | 24% | 17 | 18% | 21 | 19% |
| Failure to replicate on all 5 criteria | 2 | 11% | 5 | 15% | 21 | 22% | 21 | 19% |
| Total | 19 |  | 33 |  | 97 |  | 112 |  |
|  |  |  |  |  |  |  |  |  |
| **ORIGINAL NULL RESULTS** |  |  |  |  |  |  |  |  |
| Successful replication on all five criteria | 4 | 36% | 5 | 42% | 6 | 40% | 6 | 30% |
| Success on 4, Failure on 1 | 2 | 18% | 2 | 17% | 2 | 13% | 2 | 10% |
| Success on 3, Failure on 2 | 2 | 18% | 2 | 17% | 3 | 20% | 1 | 5% |
| Success on 2, Failure on 3 | 3 | 27% | 3 | 25% | 3 | 20% | 4 | 20% |
| Success on 1, Failure on 4 | 0 | 0% | 0 | 0% | 0 | 0% | 5 | 25% |
| Failure to replicate on all 5 criteria | 0 | 0% | 0 | 0% | 1 | 7% | 2 | 10% |
| Total | 11 |  | 12 |  | 15 |  | 20 |  |

Five of the criteria we used to assess replications could be used for both positive results and null results. The number of papers, experiments, effects, and outcomes where replications were successful on various numbers of these criteria are shown for positive results (top) and null results (bottom). The five criteria were: i) direction and statistical significance (*p* < 0.05); ii) original effect size in replication 95% confidence interval; iii) replication effect size in original 95% confidence interval; iv) replication effect size in original 95% prediction interval; v) meta-analysis combining original and replication effect sizes is statistically significant (*p* < 0.05). The data in this table are based on native effect sizes (where possible). Summarized effect-level metrics at the experiment and paper level were obtained by calculating percentages to summarize binary metrics of replication success, and by using harmonic mean *p*-values to summarize the continuous metric.

**Table S10. Number of replications inconsistent with original experiments across five replication criteria that render dichotomous outcome assessments summarizing at the effect-level with SMD effect sizes.**

|  | Papers | | Experiments | | Effects | | All outcomes | |
| --- | --- | --- | --- | --- | --- | --- | --- | --- |
|  |  |  |  |  |  |  |  |  |
| **ORIGINAL POSITIVE RESULTS** |  |  |  |  |  |  |  |  |
| Successful replication on all 5 criteria | 3 | 16% | 3 | 9% | 13 | 13% | 20 | 18% |
| Success on 4, Failure on 1 | 3 | 16% | 8 | 24% | 15 | 15% | 13 | 12% |
| Success on 3, Failure on 2 | 2 | 11% | 1 | 3% | 11 | 11% | 13 | 12% |
| Success on 2, Failure on 3 | 4 | 21% | 9 | 27% | 22 | 23% | 26 | 23% |
| Success on 1, Failure on 4 | 5 | 26% | 7 | 21% | 15 | 15% | 19 | 17% |
| Failure to replicate on all 5 criteria | 2 | 11% | 5 | 15% | 21 | 22% | 21 | 19% |
| Total | 19 |  | 33 |  | 97 |  | 112 |  |
|  |  |  |  |  |  |  |  |  |
| **ORIGINAL NULL RESULTS** |  |  |  |  |  |  |  |  |
| Successful replication on all 5 criteria | 6 | 55% | 7 | 58% | 7 | 47% | 6 | 30% |
| Success on 4, Failure on 1 | 1 | 9% | 1 | 8% | 2 | 13% | 2 | 10% |
| Success on 3, Failure on 2 | 2 | 18% | 2 | 17% | 3 | 20% | 5 | 25% |
| Success on 2, Failure on 3 | 2 | 18% | 2 | 17% | 2 | 13% | 2 | 10% |
| Success on 1, Failure on 4 | 0 | 0% | 0 | 0% | 0 | 0% | 3 | 15% |
| Failure to replicate on all 5 criteria | 0 | 0% | 0 | 0% | 1 | 7% | 2 | 10% |
| Total | 11 |  | 12 |  | 15 |  | 20 |  |

Five of the criteria we used to assess replications could be used for both positive results and null results. The number of papers, experiments, effects, and outcomes where replications were successful on various numbers of these criteria are shown for positive results (top) and null results (bottom). The five criteria were: i) direction and statistical significance (*p* < 0.05); ii) original effect size in replication 95% confidence interval; iii) replication effect size in original 95% confidence interval; iv) replication effect size in original 95% prediction interval; v) meta-analysis combining original and replication effect sizes is statistically significant (*p* < 0.05). The data in this table are based on standardized mean difference (SMD) effect sizes. Summarized effect-level metrics at the experiment and paper level were obtained by calculating percentages to summarize binary metrics of replication success, and by using harmonic mean *p*-values to summarize the continuous metric.

**Table S11. Pairwise metrics of replication success aggregated at the experiment level using SMD effect sizes.**

| Paper and Experiment ID | # of Effects | Original ES | Replication ES | Meta-analysis ES | Replication ES in PI | Replication ES in PI (sensitivity) | *P_orig_* | *P_orig_* (sensitivity) | Direction and statistical significance | Expected percent significance agreement | Expected significance agreement (sensitivity) |
| --- | --- | --- | --- | --- | --- | --- | --- | --- | --- | --- | --- |
| p15e1 | 1 | 7.41 | -0.32 | 0 | 0 (0%) | 0 (0%) | 0.028 | 0.028 | 0 (0%) | 96% | 96% |
| p15e2 | 1 | 1.61 | 0.22 | 0.86 | 1 (100%) | 1 (100%) | 0.081 | 0.091 | 0 (0%) | 76% | 74% |
| p15e3 | 1 | 2.30 | 0.25 | 1.26 | 0 (0%) | 0 (0%) | 0.013 | 0.016 | 0 (0%) | 92% | 91% |
| p16e3 | 3 | 6.50 | 3.31 | 4.13 | 2 (67%) | 2 (67%) | 0.1 | 0.1 | 3 (100%) | 95% | 95% |
| p19e1 | 2 | 2.45 | 14.32 | 3.07 | 0 (0%) | 0 (0%) | 0.017 | 0.017 | 2 (100%) | 9% | 9% |
| p19e2 | 2 | 1.43 | 0.40 | 0.82 | 0 (0%) | 1 (50%) | 0.033 | 0.055 | 1 (50%) | 97% | 90% |
| p1e2 | 5 | 13.95 | 1.10 | 1.40 | 1 (20%) | 1 (20%) | 0.0067 | 0.0068 | 3 (60%) | 99% | 99% |
| p1e3 | 6 | 3.63 | 1.48 | 1.87 | 6 (100%) | 6 (100%) | 0.16 | 0.16 | 4 (67%) | 85% | 85% |
| p1e5 | 1 | 4.58 | -0.37 | 0.53 | 0 (0%) | 0 (0%) | 0.011 | 0.011 | 0 (0%) | 94% | 93% |
| p1e6 | 1 | 16.21 | 7.55 | 9.20 | 1 (100%) | 1 (100%) | 0.16 | 0.16 | 1 (100%) | 96% | 96% |
| p20e1 | 2 | 1.69 | 0.03 | 0.47 | 0 (0%) | 1 (50%) | 0.0058 | 0.008 | 0 (0%) | 85% | 83% |
| p20e2 | 3 | 1.97 | 0.24 | 0.87 | 1 (33%) | 1 (33%) | <0.0001 | 0.00031 | 0 (0%) | 91% | 89% |
| p21e1 | 2 | 2.18 | 1.10 | 1.44 | 2 (100%) | 2 (100%) | 0.18 | 0.18 | 2 (100%) | 86% | 85% |
| p24e1 | 4 | 5.28 | 0.71 | 1.38 | 2 (50%) | 2 (50%) | 0.021 | 0.023 | 1 (25%) | 93% | 93% |
| p24e2 | 6 | 4.78 | -2.36 | -0.97 | 0 (0%) | 0 (0%) | <0.0001 | <0.0001 | 0 (0%) | 93% | 93% |
| p24e3 | 7 | 8.03 | 3.14 | 3.79 | 5 (71%) | 5 (71%) | 0.062 | 0.063 | 5 (71%) | 84% | 84% |
| p24e4 | 9 | 5.57 | 1.48 | 1.97 | 7 (78%) | 7 (78%) | 0.033 | 0.035 | 5 (56%) | 90% | 90% |
| p28e2 | 1 | 1.22 | -0.17 | 0.36 | 1 (100%) | 1 (100%) | 0.11 | 0.12 | 0 (0%) | 58% | 57% |
| p28e3 | 2 | 1.52 | 0.14 | 0.23 | 2 (100%) | 2 (100%) | 0.12 | 0.13 | 0 (0%) | 74% | 74% |
| p29e2 | 2 | 21.67 | 12.19 | 13.99 | 2 (100%) | 2 (100%) | 0.31 | 0.31 | 2 (100%) | 88% | 88% |
| p29e3 | 1 | 0.42 | 0.07 | 0.18 | 1 (100%) | 1 (100%) | 0.069 | 0.21 | 0 (0%) | 86% | 72% |
| p37e1 | 1 | 2.96 | 0.53 | 1.01 | 0 (0%) | 0 (0%) | 0.01 | 0.012 | 0 (0%) | 99% | 98% |
| p39e1 | 1 | 2.54 | -1.13 | -0.16 | 0 (0%) | 0 (0%) | 0.0032 | 0.0037 | 0 (0%) | 85% | 84% |
| p42e2 | 4 | 2.88 | 0.37 | 0.97 | 2 (50%) | 2 (50%) | 0.0092 | 0.0099 | 0 (0%) | 87% | 86% |
| p44e1 | 4 | 0.34 | -0.02 | 0.10 | 1 (25%) | 4 (100%) | 0.011 | 0.14 | 0 (0%) | 85% | 69% |
| p47e1 | 10 | 1.83 | 0.28 | 0.58 | 8 (80%) | 8 (80%) | 0.039 | 0.046 | 0 (0%) | 89% | 87% |
| p48e1 | 1 | 4.19 | 1.56 | 2.03 | 1 (100%) | 1 (100%) | 0.15 | 0.15 | 1 (100%) | 93% | 93% |
| p48e2 | 3 | 0.27 | 0.17 | 0.20 | 1 (33%) | 3 (100%) | 0.00031 | 0.57 | 3 (100%) | 100% | 77% |
| p50e1 | 1 | 0.50 | 0.43 | 0.48 | 1 (100%) | 1 (100%) | 0.7 | 0.79 | 1 (100%) | 82% | 69% |
| p5e1 | 2 | 84.23 | 2.51 | 2.61 | 0 (0%) | 0 (0%) | 0.025 | 0.025 | 2 (100%) | 99% | 99% |
| p6e1 | 1 | 6.41 | 4.32 | 4.80 | 1 (100%) | 1 (100%) | 0.55 | 0.55 | 1 (100%) | 81% | 81% |
| p9e2 | 5 | 1.76 | 0.81 | 1.01 | 5 (100%) | 5 (100%) | 0.099 | 0.12 | 5 (100%) | 97% | 95% |
| p9e3 | 2 | 1.10 | 0.33 | 0.66 | 2 (100%) | 2 (100%) | 0.39 | 0.4 | 0 (0%) | 38% | 39% |

Results of sensitivity analyses to determine the extent to which accounting for possible heterogeneity alters outcomes for three assessment criteria. This table contains the following columns (from left to right): paper and experiment ID (e.g., p15e1 is experiment 1 in paper 15); number of effects in that experiment; original effect size (ES); replication ES; meta-analytically combined ES; number of effects for which the replication ES is within the 95% prediction interval (PI); result of sensitivity analysis for previous column; *P_orig_*; result of sensitivity analysis for *P_orig_*; direction and statistical significance; expected percent significance agreement; result of sensitivity analysis for previous column. The sensitivity analyses yielded similar results to the main analyses likely because the estimated heterogeneity was small relative to the original and replication standard errors. The total number of effects is 97.
